# Loading of the hip and knee joints during whole body vibration training

**DOI:** 10.1371/journal.pone.0207014

**Published:** 2018-12-12

**Authors:** Georg Bergmann, Ines Kutzner, Alwina Bender, Jörn Dymke, Adam Trepczynski, Georg N. Duda, Dieter Felsenberg, Philipp Damm

**Affiliations:** 1 Julius Wolff Institute, Charité – Universitätsmedizin Berlin, Berlin, Germany; 2 Center for Muscle and Bone Research, Charité – Universitätsmedizin Berlin, Berlin, Germany; University of L'Aquila, ITALY

## Abstract

During whole body vibrations, the total contact force in knee and hip joints consists of a static component plus the vibration-induced dynamic component. In two different cohorts, these forces were measured with instrumented joint implants at different vibration frequencies and amplitudes. For three standing positions on two platforms, the dynamic forces were compared to the static forces, and the total forces were related to the peak forces during walking. A biomechanical model served for estimating muscle force increases from contact force increases. The median static forces were 122% to 168% (knee), resp. 93% to 141% (hip), of the body weight. The same accelerations produced higher dynamic forces for alternating than for parallel foot movements. The dynamic forces individually differed much between 5.3% to 27.5% of the static forces in the same positions. On the Powerplate, they were even close to zero in some subjects. The total forces were always below 79% of the forces during walking. The dynamic forces did not rise proportionally to platform accelerations. During stance (Galileo, 25 Hz, 2 mm), the damping of dynamic forces was only 8% between foot and knee but 54% between knee and hip. The *estimated* rises in muscle forces due to the vibrations were in the same ranges as the contact force increases. These rises were much smaller than the vibration-induced EMG increases, reported for the same platform accelerations. These small muscle force increases, along with the observation that the peak contact and muscle forces during vibrations remained far below those during walking, indicate that dynamic muscle force *amplitudes* cannot be the reason for positive effects of whole body vibrations on muscles, bone remodelling or arthritic joints. Positive effects of vibrations must be caused by factors other than raised forces amplitudes.

## Introduction

**Whole body vibration (*WBV*)** training induces vibrations with low strokes (total movement) and high frequencies at both feet during stance. One aim of WBV can be to exercise the muscles with less effort than required for conventional training. Many, but not all, studies report positive training effects (Supplement 1). However, it is unclear whether positive results are caused by high, vibration-induced muscle forces or by other mechanisms. With instrumented joint implants, the **joint contact forces** (***JCF***) during the vibrations were telemetrically measured and compared to those without vibrations in the same position and during normal walking. A simplified biomechanical model allowed us to estimate the vibration-induced *muscle* forces from the measured JCF to answer this open question.

### Possible effects of whole body vibrations

Different mechanisms have been described concerning how muscles may be affected by WBV. A detailed overview can be found in [1]. The change in a muscle’s length, caused by vibrations, can cause a stretch reflex, which activates the muscle spindles [2, 3]. However, it was also shown that vibrations do not potentiate muscle spindle function as hypothesized [4]. It has further been suspected that muscles are activated, and thus trained, by shifting the resonance frequency of body parts away from the frequency of the external vibrations, such reducing oscillating strokes and discomfort [5, 6]. WBV may also inhibit antagonistic muscle activities [1], increase the blood flow in the lower legs [7], induce the switch from Type 1 to Type 2 muscle fibres [8] or increase the concentration of testosterone and growth hormones [9].

*Another* possible reason for an effective muscle training by WBV may be if the muscle forces *magnitudes* during the vibrations are very high. We hypothesize that they must then be higher than during everyday activities. For checking this possibility, we compare the measured joint contact forces during WBV to those during the frequent activity ‘walking’ (other everyday activities even cause higher forces [10, 11]). A new biomechanical model allowed to derive the muscle forces, required for this comparison, from the joint contact forces.

### Relations between accelerations and joint contact forces

During WBV training, a subject with the **body mass m** stands on a vibrating platform that applies vertical sinusoidal movements to both feet, either in the same or in opposite directions. Typical **peak-to-peak strokes** (***s***) and **frequencies** (***f***) are 1 to 5 mm and 10 to 50 Hz. The **peak acceleration at the foot (*a***_***foot***_**)** is then:

$$a_{foot}=\left(\frac{s}{2}\right)*{\left(2*\pi *f\right)}^{2}$$ (Eq 1)

It may be a multiple of the gravitation acceleration g (e.g., a_foot_ = 3.4 g at f = 25 Hz and s = 2.5 mm).

If the whole body (*mass* ***m***) were absolutely rigid and fixed to the platform, the ***hypothetical* dynamic force between the foot and the platform** (***F’***_***foot***_) in the case of parallel vertical foot movements would be:

$${F^{\prime }}_{foot}=a_{foot}*\frac{m}{2}=s*m*\pi^{2}*f^{2}$$ (Eq 2)

This force would act additionally to the static force (g*m/2) at each foot. In reality, the elasticity of bones, muscle-controlled joint flexions, damping by muscles and ‘wobbling masses’ of the upper body [12] have a frequency-dependent damping effect, which reduces the theoretical value of F’_foot_ to the ***real* dynamic force at the foot (*F***_***foot***_). On the way from the foot to the knee and hip joints, the local acceleration is further reduced in dependency from, among other factors, body position and f. The acceleration was reported to be 40% of a_foot_ at the knee and 10% at the hip joint [13], or even less [14]. Due to these reduced accelerations, the contact forces in the knee and hip joints must be expected to be lower than F’_foot_.

The only two in vivo measurements on the influence of vibrations on forces in the skeleton were performed with instrumented implants in the lumbar spine. On a vibrating chair (f = 0.3–30 Hz, a = 0.1 g) the forces increased by 17–84% [15]. When standing on a vibrating platform (s = 1 and 2 mm, f = 12.5 and 25 Hz) at different body positions, the highest increase was 38% [16]. Whether higher strokes increased or decreased the force depended on the body position.

### Literature survey

A survey of the numerous publications about WBV training is given in *Supplement 1*. The search included reports of positive, negative or non-existent effects of WBV for the lower extremities in the following application areas: muscles and movements, EMG activities, bone remodelling, joint implants and osteoarthritis. For all areas this survey delivered controversial results. The effect of WBV on training the strength of the lower extremity muscles was, for example, confirmed by three meta-studies (MS), denied by four MS and found to be controversial by another two MS.

The controversial reports on all possible applications of WBV may be due to differences between investigated subjects and applied vibration parameters (f, s, platform type). It may also be that other parameters (e.g., number of motor neurons, neuronal-muscle-interfaces, muscle energy supply, glycogen resources, numbers of mitochondria in muscle cells, activity of satellite cells, etc.) and mechanisms were not identified yet. In none of the studies were proportional increases of EMG signals and muscle forces or JCF explicitly stated. The relation between both would have to be known to judge the possible training effects from captured EMG data.

Of special interest for our own study was this report [17]: In elderly subjects, a linear relationship between the acceleration a_foot_ and the summed EMG signals from six muscles in the lower leg was found. During ‘relaxed standing’ with a_foot_ = 50 m/s^2^, the EMG signals were approximately 100% higher than without vibrations. Our own measurements were performed at the same acceleration and allow comparison between the *measured* increase in contact forces and the *reported* increase in EMG signals.

### In vivo measurements of knee and hip contact forces

In *hip* joints, telemetric JCF measurements with instrumented implants were first published in [18, 19]. Our own group reported such measurements in 17 subjects since 1993 [10, 20–26]. For *knee* joints, resultant forces at the tibial tray [27] and spatial forces and moments from up to three patients were published by others [28]. Our own group made data from nine subjects public [11, 29–39]. Measured JCF and synchronous patient videos during many different activities can be accessed in the public database OrthoLoad.com [40].

### Goals of this study

The JCFs in knee and hip joints were measured in vivo during WBV training with the goals of

1. collecting basic data on the induced dynamic joint contact forces,
2. estimating the muscle forces from the contact forces to answer the question of whether the dynamic muscle forces are high enough to generate training effects.

## Material and methods

In vivo measurements of JCF during WBV were performed in two different cohorts of patients with instrumented knee and hip implants. The measurement setups and evaluation methods were identical.

### Instrumented implants

An Innex total *knee* implant (Zimmer GmbH. Winterthur) was instrumented with an inductive power supply, load sensors and telemetric data transfer [41] to measure the three force and three moment components of the contact load that acts on the tibial tray [42] at asynchronous frequencies of approximately 100 Hz. The **resultant force** (***F***) was calculated from its three components. Because the moments only contain information about friction in the joints, they are not reported here. The deviation of the peak forces from the tibial axis is small during stance/squat (6°/7°) [11]. Therefore, the force component acting in the axis direction is nearly identical to the resultant force F.

A CTW total *hip* implant (Merete Medical, Berlin) was instrumented with the same electronics [41] to measure the six load components acting at the implant head [25]. F deviated from the long axis of the femur by 16°/24° during stance/squat [10]. Therefore, the force component in the direction of the femur axis is typically only 4%/9% smaller than the resultant force F, reported here.

### Investigated subjects

Measurements were taken in knee and hip patients from two studies which were approved by the Charité Ethics Committee (knee: EA4/069/06, hip: EA2/057/09) and registered at the German Clinical Trials Register (knee: DRKS00000606, hip: DRKS00000563). All patients gave written informed consent prior to participating in these studies and having their images published.

Six knee patients with primary gonarthrosis (Table 1) and four hip patients with primary coxarthrosis (transgluteal approach) were included in this study. On average, measurements were taken 39 (knee) and 14 (hip) months after joint replacement. All subjects exercised on the vibration platforms without restrictions or pain.

**Table 1:** Subject data. The measurements at the knee and hip were taken in two different cohorts. Postop. Time = Time of measurements.

**Knee Joint**							
Subject [abbreviation]	**K1**	**K3**	**K5**	**K7**	**K8**	**K9**	**Mean**
Sex [male/female]	m	m	m	f	m	m	
Age [years]	66	74	63	77	73	77	71.7
Body Weight [kg]	98.5	98.0	92.2	67.9	77.0	110.0	90.6
Postop. Time [months]	46	45	39	37	33	31	38.5
**Hip Joint**							
Subject [Abbreviation]	**H2**	**H3**	**H4**	**H5**			**Mean**
Sex [male/female]	m	m	m	f			
Age [years]	63	61	51	63			59.5
Body Weight [kg]	82.5	90.3	81.1	87.9			85.5
Postop. Time [months]	18	15	12	10			13.8

### Measurements

Measurements were taken at different frequencies f, strokes s and body positions (Table 2).

**Table 2:** Measurement conditions. The vibration strokes s = 2 to 4 mm are peak-to-peak values. The force F_foot_ between platform and foot was only measured on the GalileoS. Walking at 4 km/h and stance without vibrations in the three positions were additionally investigated.

Body Position and Activities	Knees 15° Flexed	Knees 50° Flexed	Forefoot Stance
**GalileoS**	25 Hz, 2.5 mm	25 Hz, 2.5 mm	25 Hz, 2.5 mm
	F_foot_ measured	F_foot_ measured	F_foot_ measured
**Galileo**	12.5 Hz, 2 + 4 mm	---	---
	25 Hz, 2 + 4 mm	---	---
**Powerplate**	25 Hz, 2 + 4 mm	---	---
	50 Hz, 2 + 4 mm	---	---

#### Vibrating platforms

Three platform types were used:

**Galileo 2000 (*Galileo*)**: This platform (Novotec Medical and Stratec Medizintechnik, Germany) induced opposite vertical foot movements. The vibration frequency and the stroke (total platform movement) were exactly determined by the platform construction.

**Galileo 2000 Sensor (*GalileoS*)**: This platform was identical to Galileo, but additional transducers allowed for measurement of the forces F_foot_, acting at the foot.

**Powerplate 5 (*Powerplate*)**: This platform (Powerplate GmbH, Germany) induced parallel vertical foot movements. Due to its design, stroke and frequency slightly depended on the subject’s body weight; they were not controlled.

#### Body positions and activities

**Fig 1:**
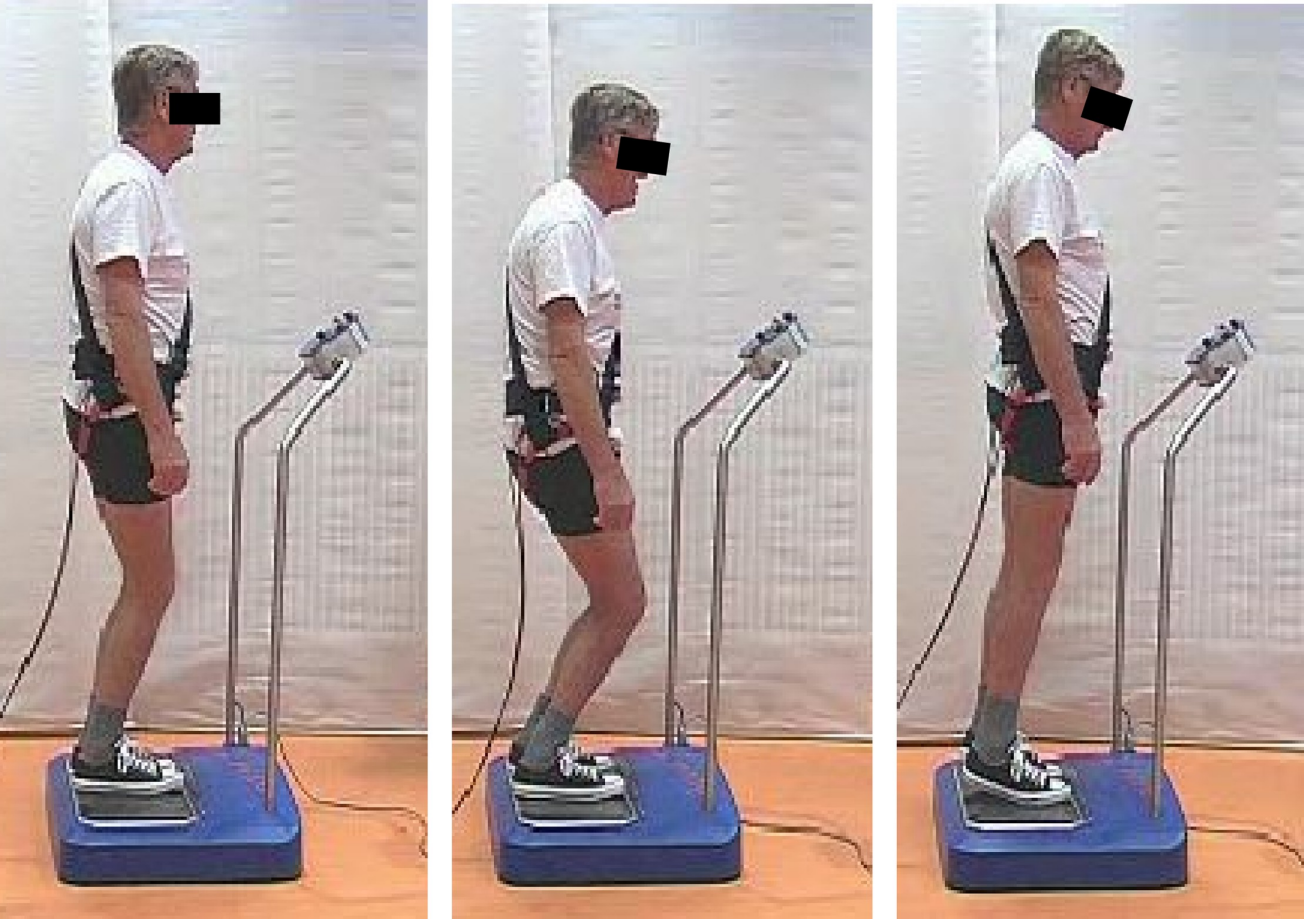
Subject in three positions on Powerplate. From left: Knees 15° flexed, knees 50° flexed, forefoot stance.

Three different body positions were investigated, two of them only on the GalileoS (Table 2). The body positions (Fig 1) were controlled by an experienced physiotherapist. The vibration exercises lasted for 13 to 20 seconds.

**Knees 15° flexed**: Upright stance with 10° to 20° knee flexion and whole-foot support.

**Knees 50° flexed**: Upright stance with 45° to 55° knee flexion and whole-foot support.

**Forefoot stance**: Upright stance on the forefeet with straight knees.

Two activities were additionally investigated:

**Stance**: F was measured for 20 s without vibrations with the knees 15° flexed.

**Walking**: Three minutes walking at 4 km/h on a treadmill.

### Evaluation of data

Separately for the knee and hip cohorts, all forces were calculated for each subject in **percent of the body weight** (***%BW***) and then averaged (median and range) across the subjects. All dynamic force values refer to the amplitudes (zero to peak). The influence of parameter changes (f, s, platform type, body position) on the dynamic contact forces was analysed statistically, using the Wilcoxon rank sum test. ‘Significance’ and ‘low significance’ were defined as p ≤ 0.05 and p ≤ 0.10, respectively.

#### Static and dynamic joint forces during vibrations

**Fig 2:**
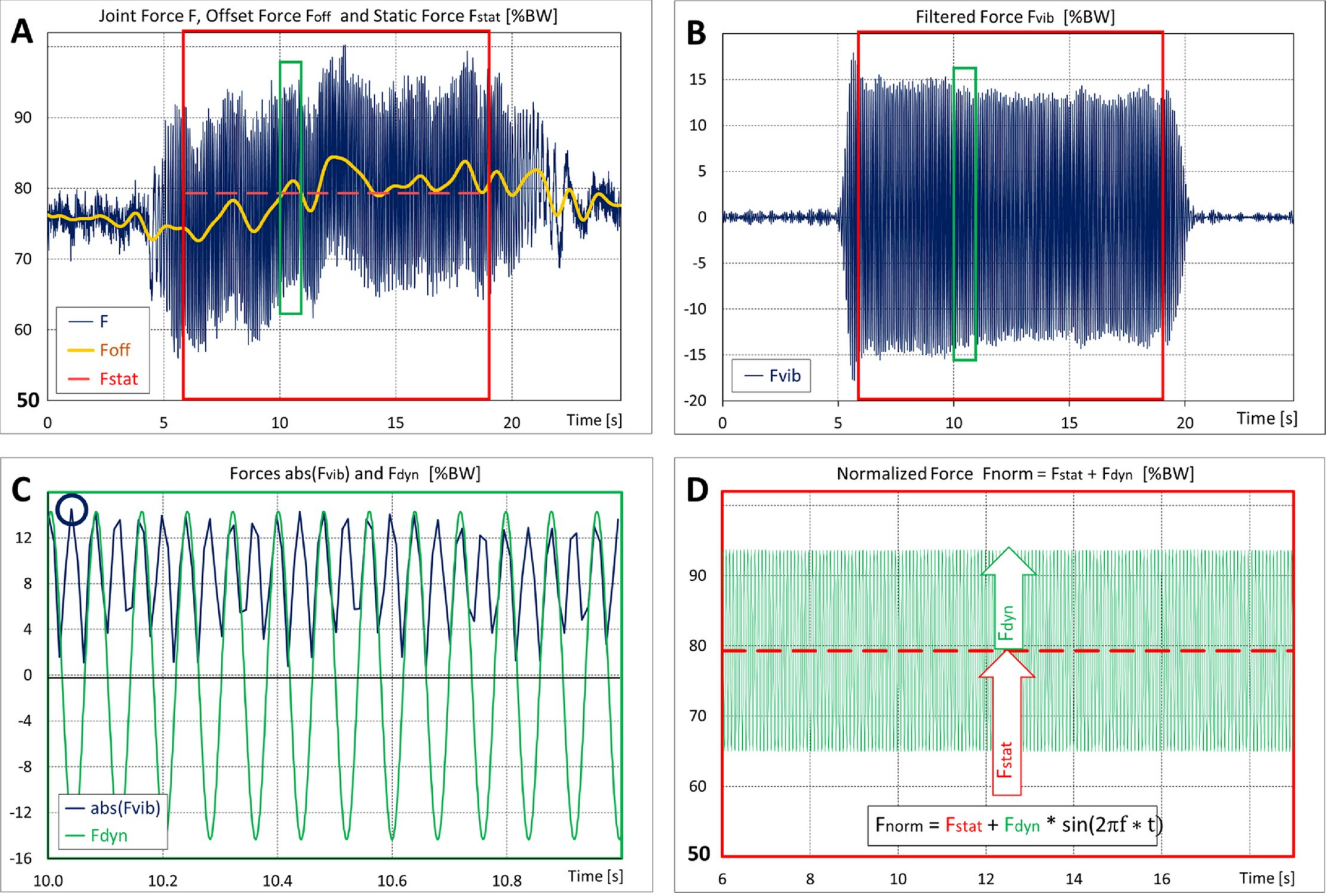
Measured contact force and data evaluation. Data from the preceding feasibility study. Galileo, knees 15° flexed, 12.5 Hz, 2.5mm. Vibrations between 4.5 s and 22 s. Evaluation range 6 s to 19 s (red frames). Note that the scales in A and D start at 50%BW. **A**: The joint force F consists of the offset force F_off_ (yellow), the vibration component F_vib_ around F_off_ (blue) and small noise. F_off_ is obtained by lowpass filtering of F. F_stat_ (red) see explanation for D. **B**: Bandpass filtering of F eliminates F_off_ plus noise and delivers F_vib_. **C**: Detail from the green boxes in A, B. F_vib_ is rectified (abs(F_vib_), blue). Every second, the maximum is determined (blue circle), and all maxima are averaged. This averaging delivers the amplitude F_dyn_ of the dynamic force (green). **D**: Averaging of F_off_ delivers the static preload F_stat_ (dashed red). Reported are the median values and inter-individual ranges of F_stat_ and F_dyn_ of the ‘normalized’ joint force F_norm_ = F_stat_ + F_dyn_ * sin(2πf * t).

F contained three components (Fig 2A, blue):

- the **offset force** (***F***_***off***_**),** which depended on the slightly varying body position and possibly also on antagonistic muscle activities,
- the **vibration force (*F***_***vib***_) and
- small noise due to the 4 kHz frequency of the power supply.

Time intervals were evaluated when F_vib_ was nearly stationary (red boxes), using a customized programme (Matlab R2011a). Fast Fourier transformations showed relevant amplitudes at f and below 3 Hz. Low pass filtering of F (*Butterworth order 10*, *cut-off frequency 4 Hz)* delivered F_off_ (Fig 2A, yellow). The **average static force** (***F***_***stat***_) was then calculated as the arithmetic mean of F_off_ during the evaluated time (Fig 2D, dashed red).

Bandpass filtering of F *(Butterworth order 10*, *centre frequency f*, *bandwidth ± 2*.*7 Hz)* resulted in F_vib_ (Fig 2B). Because the asynchronous, force-dependent sampling frequency of approximately 100 Hz was in the order of magnitude of the vibration frequencies of 12.5 to 50 Hz, the dynamic force amplitude could not be obtained accurately during every vibration cycle. Therefore, F_vib_ was rectified (Fig 2C, blue) and its maxima were determined every second (example: blue circle). All maxima were averaged, which delivered the amplitude of the **average dynamic peak force (*F***_***dyn***_) for a given subject (Fig 2C and 2D, green).

These procedures lead to the **‘normalized’ contact force (*F***_**norm)**_ in the joint:

$$F_{norm}=F_{stat}+F_{dyn}*sin(2\pi f*t)$$ (Eq 3)

#### Static forces without vibration

For standing with 15° knee flexion, F_stat_ with vibrations was compared to the **maximum force without vibrations (*F***_***0***_).

#### Dynamic forces at the foot

The *measured* dynamic force F_foot_ at the foot was compared to its *hypothetic* value F’_foot_ that would act if the whole body were rigid (Eq 2). Furthermore, F_dyn_ in the knee and hip were compared to F_foot_ to access the force attenuation from the foot over the knee to the hip.

#### Peak forces during walking

F during walking was evaluated by a customized programme (Visual Basic). The time patterns of F throughout 15 single steps after two minutes of walking were averaged by a ‘dynamic time warping’ procedure which was designed as to best maintain the force maxima [43]. During the obtained average step, F always showed a ‘double-peak’ pattern within the stance phase [10, 11]. The largest of both peaks was taken as the **peak force** (***F***_***peak***_) during walking.

### Biomechanical model of the relation between contact and muscle forces

As described in the Introduction, our measured rise in contact forces during WBV can be compared to reported increases in EMG signals [17] under the same test conditions. The following biomechanical model of the knee allows to estimate the muscle forces from the measured contact forces. Using this model, the vibration-induced increase in muscle forces can be compared to the reported increase in EMG signals. This allows a decision on whether it is legitimate to conclude from rises in EMG signals during WBV on proportional increases in muscle forces.

**Fig 3:**
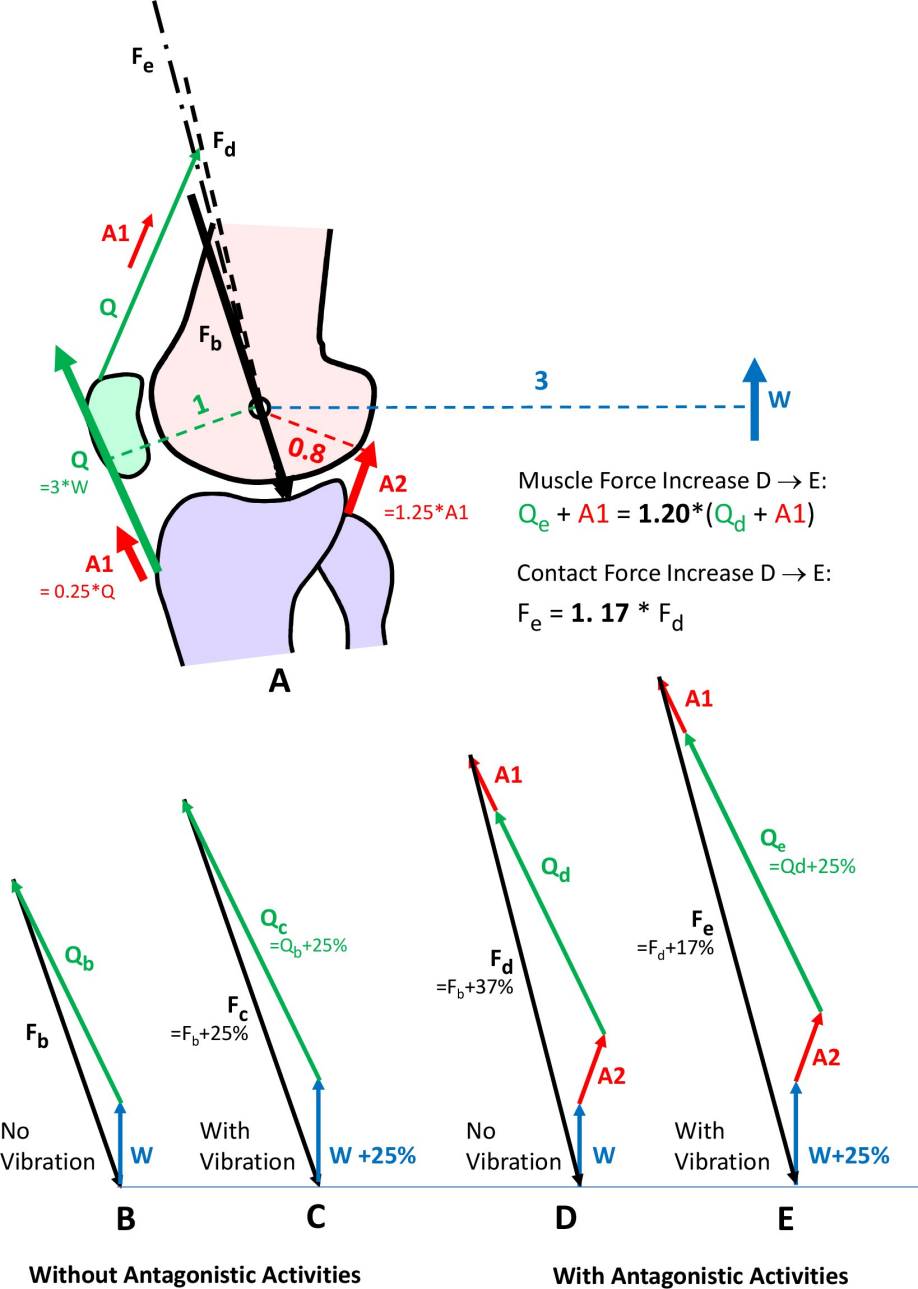
Simplified static knee model in the sagittal plane. **(A)** W = ground reaction force. Q = minimally required quadriceps force at tibia to balance W. F_b_ to F_e_ = joint contact forces. A1 = additional antagonistic component of Q, always assumed to be 25% of Q. A2 = antagonist of A1. **(B)** No antagonistic activities. **(C)** Like B, but W was increased by 25%, simulating vibrations. **(D)** With antagonistic forces A1 and A2. **(E)** Like D, but W was increased by 25%.

The simplified static model (Fig 3A) is based on the force and moment equilibrium in the sagittal plane. All forces are two-dimensional vectors. In a standing position with 15° knee flexion, the ground reaction force is only balanced by the quadriceps force. The *relative* lengths of all lever arms and the force directions were taken from the average of our knee cohort. The model describes how much contact and muscle force rise when the ground reaction force increases by 25% (simulating vibrations) while the body position remains unchanged. This model describes two cases: a) without antagonistic muscle activities (Fig 3B and 3C) and b) with a 25% increase in the quadriceps force due to antagonist activities (Fig 3D and 3E).

The following measures are used:

W Ground reaction force at foot. The lever arm of W depends on the a-p position at which the ground reaction force acts at the foot, i.e., on how much the body weight is shifted forwards or backwards.
Q Quadriceps force. The relation between W and Q depends on the relation of their lever arms. The lever arm of W was assumed to be three times larger than that of Q, resulting in Q = 3*W.
F Knee contact force

A1, A2 Additional, antagonistic muscle forces that counterbalance each other and may be exerted by the subjects for stabilizing the joint. A1 was *assumed* to always be 25% of Q_b_. A2 depends on the relation of the lever arms of A1 and A2. With the relation of 1 to 0.8, A2 becomes 25% larger than A1.

The vector of the contact force F is calculated from

F=W+Q+A1+A2

A1 and A2 are zero if antagonistic activities are lacking. This model can answer the question of what percent the muscle force |Q + A1| (length of vector Q + A1) rises for a given percentage rise of the contact force |F|.

This biomechanical model is very much simplified, since it neglects dynamic effects like damping and tendon elasticities. It can certainly only deliver rough estimates of muscle force changes. The *absolute* force values may differ from the real situation, but the *relative* force changes are more realistic.

## Results

If not mentioned otherwise, all cited forces refer to their median values. ‘Knee’ and ‘hip’ refer to the respective joint. Reductions are stated with negative signs, e.g., ‘reduction by -5%’.

### Time courses of contact force

**Fig 4:**
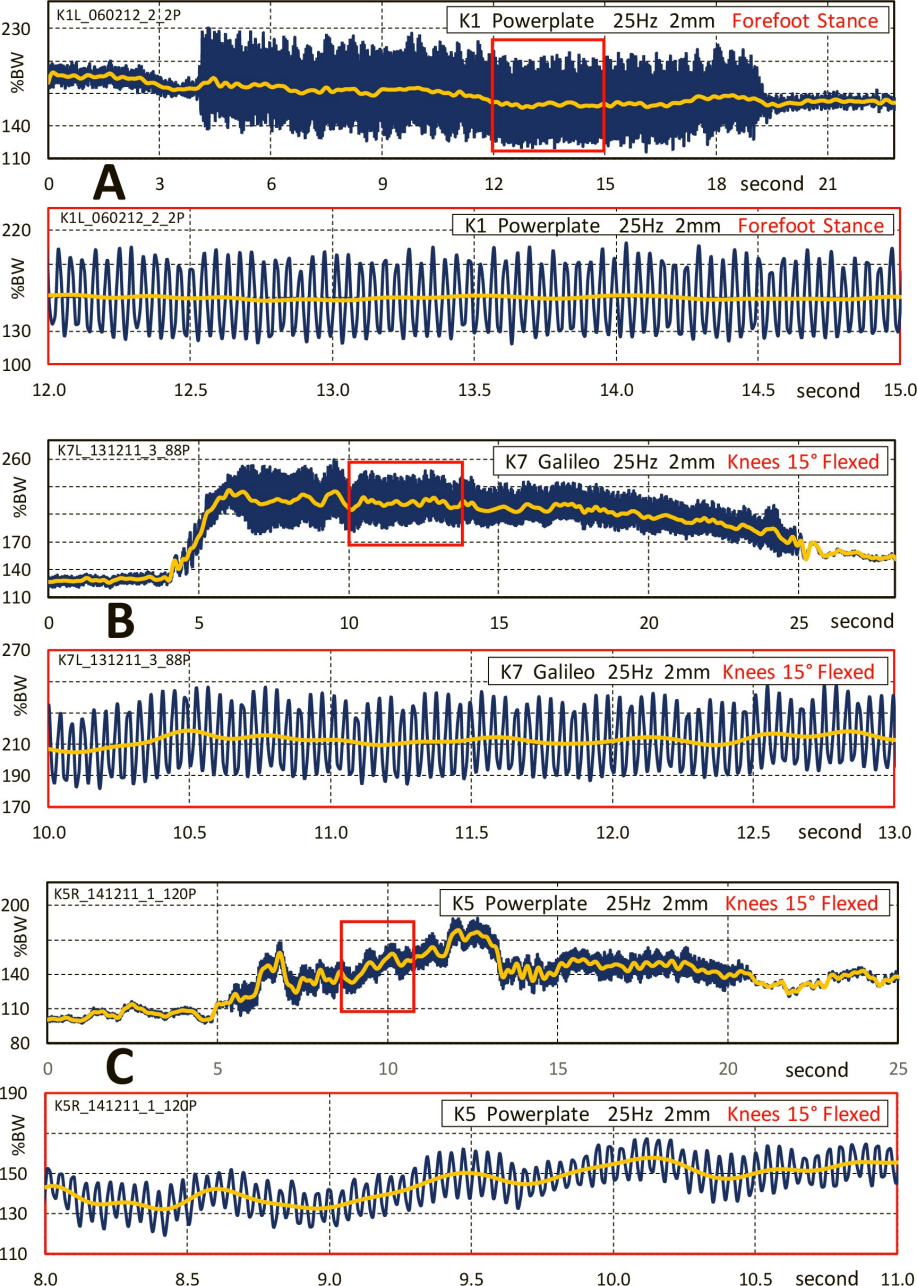
Samples of knee contact forces. A to C each contain an overview and a detail (red frames) of measurements on the Powerplate or Galileo at 25 Hz, 2 mm. Blue = contact force F. Yellow = offset force F_off_. Vibration force F_vib_ = difference between blue extrema and yellow curves. Note that the scales differ and don’t start at zero.

The contact force F not only depended on frequency, stroke and platform type but also differed substantially between the subjects (Fig 4). The offset force F_off_ was sometimes nearly constant (A), often slowly decreased or increased during the vibration time (B) and sometimes fluctuated strongly (C). The overlaid dynamic force F_vib_ had very deviating magnitudes in different individuals under the same f/s-conditions (smaller from A to C). In most cases, it stayed nearly constant (A, C), but in some subjects the dynamic amplitude became smaller with the vibration time (B).

### Dynamic forces at different frequencies, strokes and platforms

**Fig 5:**
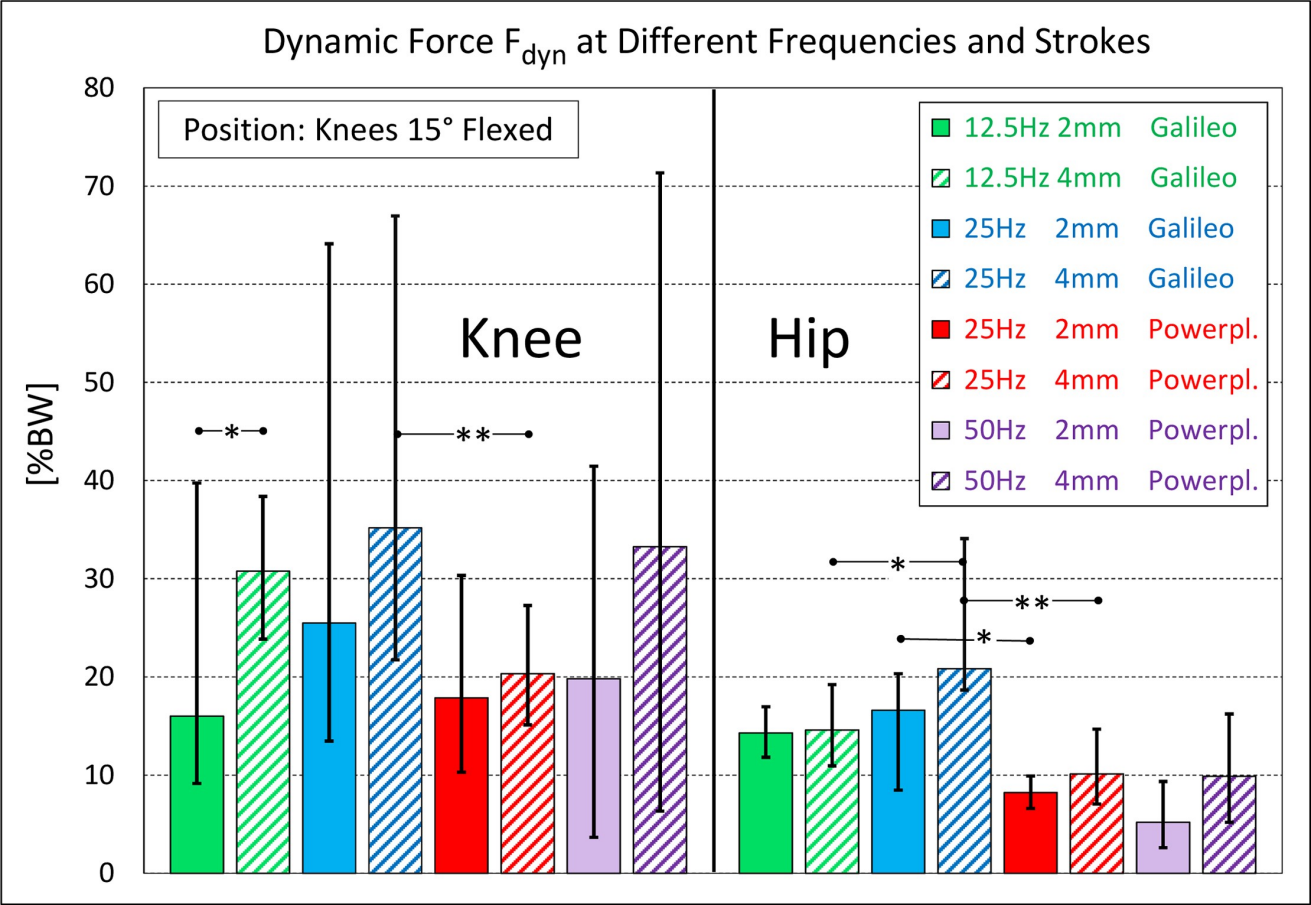
Dynamic force F_dyn_ in knee and hip joint at different frequencies and strokes. Median values and ranges on Galileo and Powerplate. Body position = knees 15° flexed. ** = significant change (p ≤ 0.05), * = low significance (p ≤ 0.10).

The dynamic forces F_dyn_ on the Galileo and Powerplate platforms at different f and s values are charted in Fig 5.

The highest median values never exceeded 35.2%BW (knee) and 20.8%BW (hip). Especially in the knee, the ranges of F_dyn_ were large, i.e., the dynamic force varied extremely between the subjects. Under some f/s-conditions the highest values were more than 100% larger than the median values, e.g., in the knee by +143% (Galileo, 25 Hz, 2 mm). On the Powerplate the upper and lower limits of F_dyn_ deviated by a factor of up to 11 (knee, 50 Hz, 4 mm). The lowest values indicate that some subjects exercised with F_dyn_ close to zero. Some subjects showed values of F_dyn_ that were close to the upper range under one f/s/platform condition but values close to the lower range under a different condition.

Under only one condition (Galileo, 12.5 Hz, 2 mm) was F_dyn_ in the knee close to that in the hip. Otherwise, F_dyn_ was always much larger in the knee. On the Galileo, this surplus was +54% to +110%; on the Powerplate, it was 101% to 283%. At 25 Hz, the median of F_dyn_ was always higher on the Galileo than that on the Powerplate. For strokes of 2 and 4 mm, this excess was +143% to +73% (knee) and +102% to +106% (hip).

However, due to the strong individual differences in F_dyn_ under all exercise conditions, reflected by the large ranges in Fig 5, only a few parameter changes caused statistically significant changes in F_dyn_. Significant changes (**, p ≤ 0.05) were only encountered in the knee and hip when the platform changed from Galileo to Powerplate (at 25 Hz, 4 mm).

### Dynamic forces at different foot accelerations

**Fig 6:**
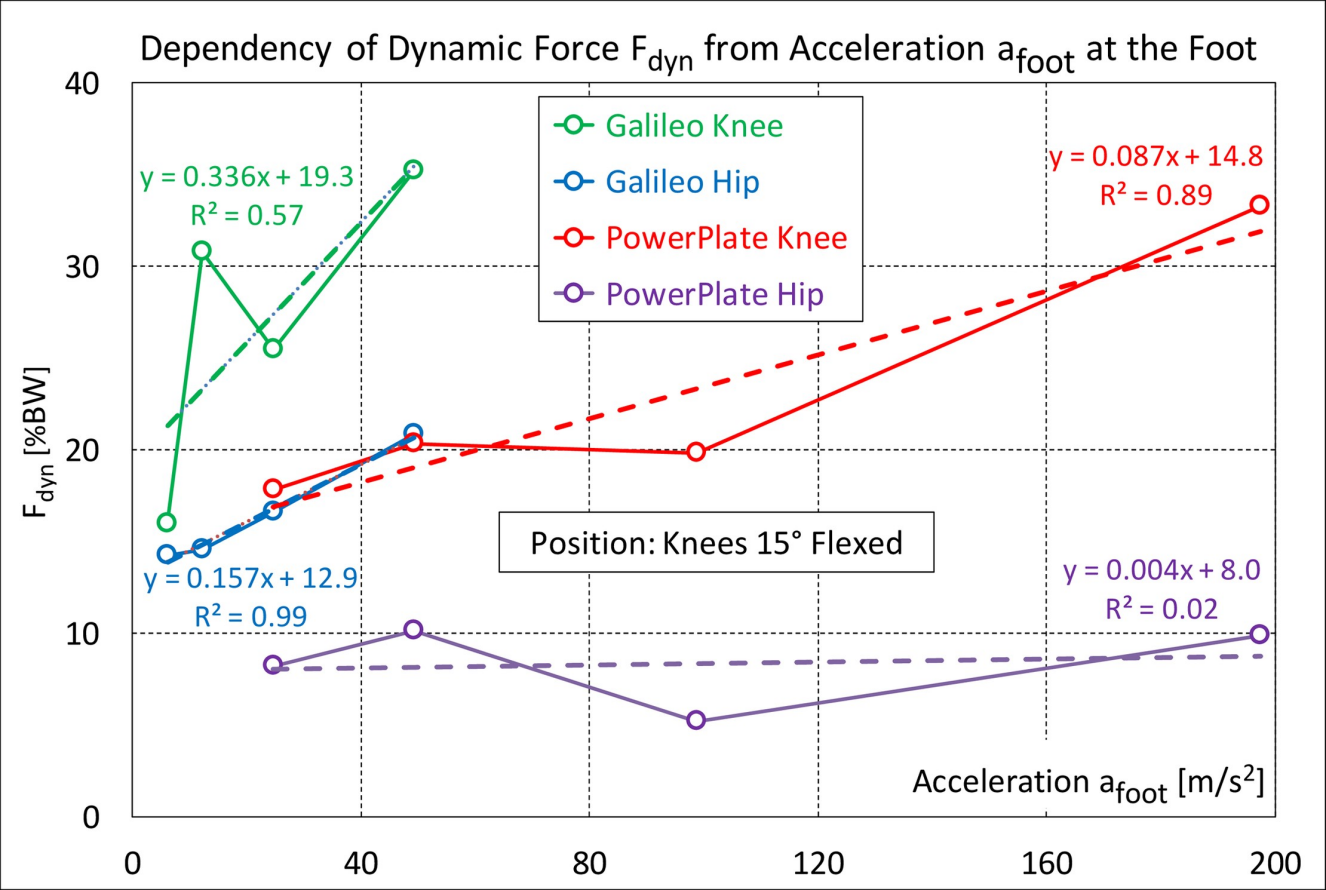
Relation between dynamic force and peak acceleration at the foot. Only for the Galileo data from the hip and the Powerplate data from the knee was a good linear correlation found between the dynamic force F_dyn_ and the acceleration a_foot_ at the foot. Because none of the correlation lines cross the zero point, F_dyn_ doesn’t change proportionally to a_foot_.

For the data in Fig 5, the platform accelerations a_foot_ (Eq 1) were 6.2 to 49.4 m/s^2^ for the Galileo and 24.7 to 197.4 m/s^2^ for the Powerplate. When charting F_dyn_ against a_foot_, very different coefficients of determination were found for the regression lines (Fig 6).

In one case R^2^ was nearly zero. For the Galileo, the R^2^ values were 0.57/0.99 (knee/hip) and for the Powerplate, they were 0.89/ 0.02. F_dyn_ and a_foot_ did not change proportionally, as none of the regression lines crossed the zero point. These regression lines would therefore predict existing dynamic forces without vibrations. Logarithmic regression functions resulted in lower R^2^ values and also predicted positive F_dyn_ values at a_foot_ = 0. The non-proportional relation between F_dyn_ and a_foot_ indicates that F_dyn_ is damped much more at higher platform accelerations than at lower ones.

### Dynamic joint forces at different body positions and comparison to dynamic forces at the foot

**Fig 7:**
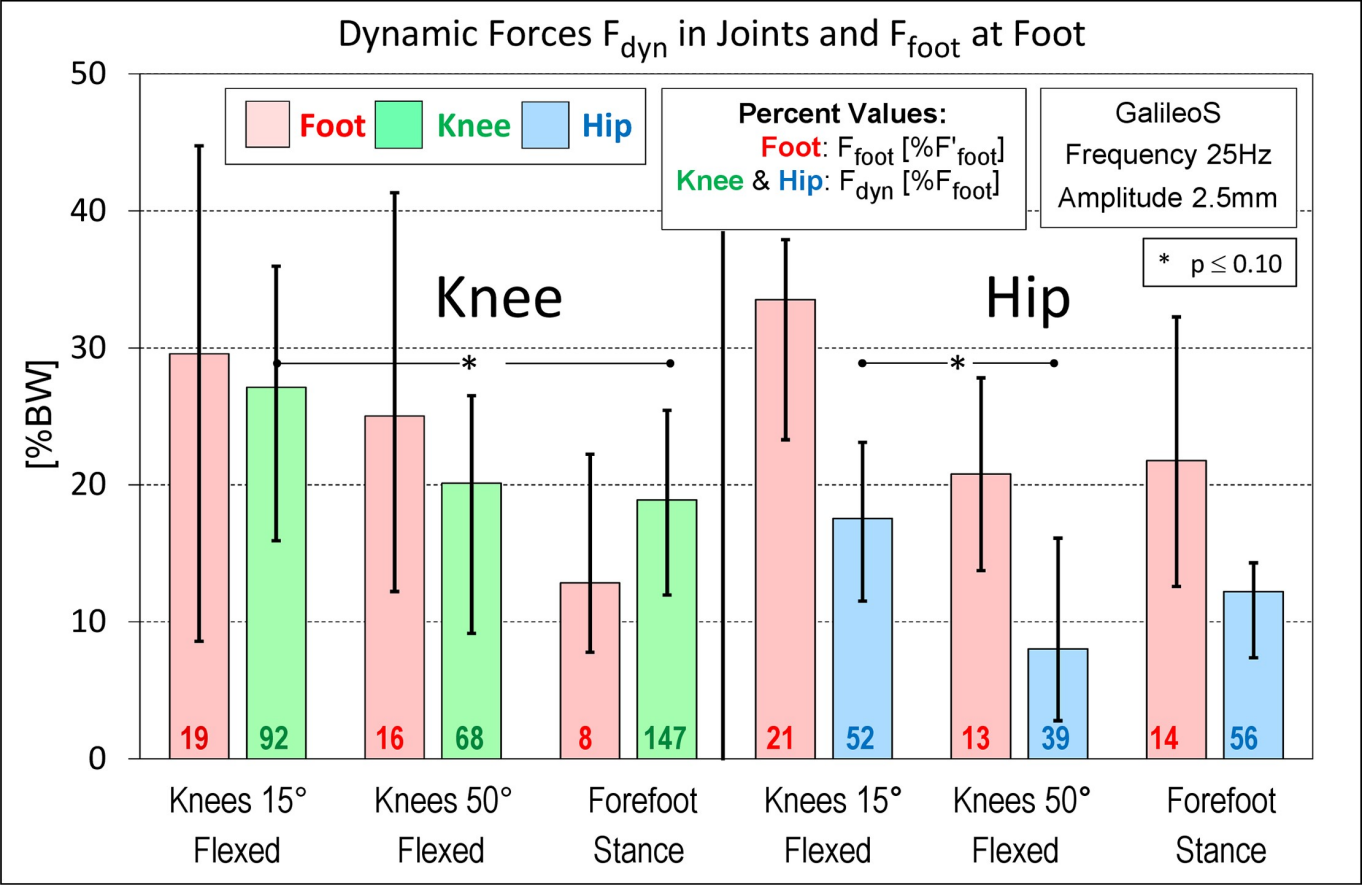
Dynamic force in the knee and hip joint in different body positions and comparisons to the dynamic force at the foot. Median values and ranges from the knee and hip joint for three different body positions. All data from GalileoS at 25 Hz, 2.5 mm. Red columns = measured force F_foot_ at foot. Green/blue columns = dynamic force F_dyn_ in the knee/hip joint. Percentage values at the bottom of columns: Red = measured force F_foot_ as a percent of the hypothetical force F’_foot_ at the foot. Green/blue = F_dyn_ as a percent of F_foot_. ** = significant change (p ≤ 0.05), * = low significance (p ≤ 0.10).

Fig 7 shows the dynamic forces F_foot_ at the foot (red columns) and the dynamic forces F_dyn_ in the knee/hip (green/blue columns) for three body positions on the GalileoS at 25 Hz and 2.5 mm. F_foot_ differs between the knee and hip because different cohorts were investigated.

For the same position, F_dyn_ was always much higher in the knee than that in the hip (+54% to +151%). With values of 27.0/17.6%BW (knee/hip), F_dyn_ was highest if the knees were flexed by only 15°. The influence of the body position on F_dyn_ was different between the knee and hip: Increasing the knee flexion to 50° reduced F_dyn_ by -26% in the knee but much more in the hip (-55%). Changing from 50° flexion to a forefoot stance slightly further decreased F_dyn_ in the knee but let it rise by +53% in the hip. As already observed for the changed parameters f/s/platform (Fig 5), the effects of changed body position on F_dyn_ were only rarely found to be significant, namely, for the knee when changing from 15° flexion to a forefoot stance and for the hip when increasing flexion from 15° to 50°.

With one exception (knee during forefoot stance), F_dyn_ in both the knee and hip were smaller than F_foot_ (green/blue percentage values). The reductions from F_foot_ to F_dyn_ were -8%/-32% in the knee for 15°/50° knee flexion, but they were more pronounced (-48%/-61%) in the hip. Unexpectedly, F_dyn_ in the knee exceeded F_foot_ by +47% when the subjects stood on the forefeet.

The forces F_foot_ also depended on the body position (Fig 7). For both joints, F_foot_ was highest if the knees were flexed by 15°. Increasing the flexion to 50° reduced F_foot_ by -16%/-38% (knee/hip). Further changing to a forefoot stance slightly reduced F_foot_ in the knee, but increased it in the hip.

F_foot_ was always much smaller (red percent values) than the force F’_foot_ (Eq 2) that would (hypothetically) act at the foot if the whole body were rigid and without any elastic or damping properties. Only 8% to 21% of F’_foot_ truly acted at the foot, with highest percentages acting if the knee was flexed by only 15°.

### Static and dynamic forces during vibrations, compared to peak forces during walking

**Fig 8:**
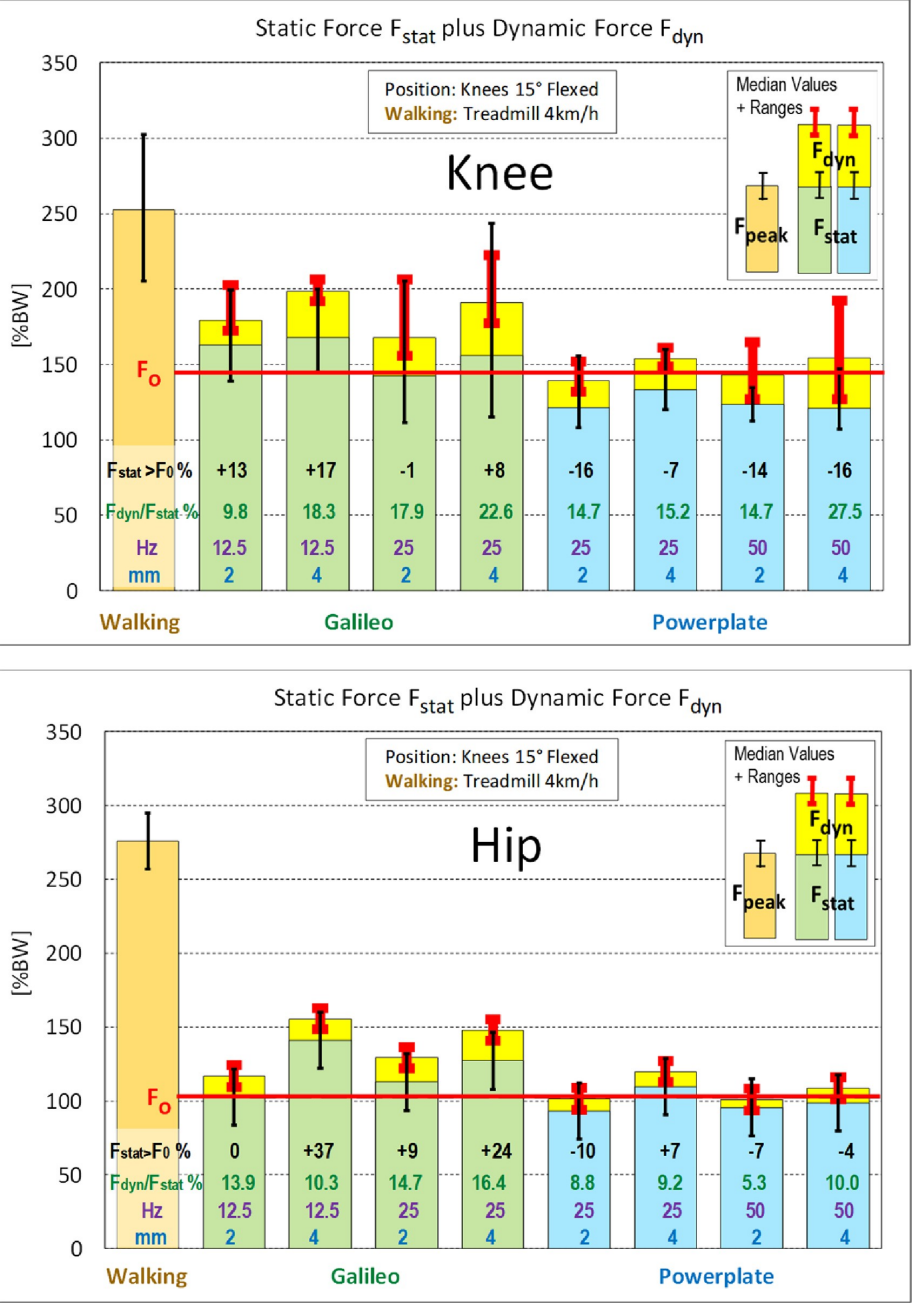
Static plus dynamic joint forces on Galileo and Powerplate platforms, compared to peak forces during walking. Body position: knees 15° flexed. Orange columns = peak force F_peak_ during walking. Green/blue columns = median plus (black) range of static force F_stat_ (with vibrations). Top yellow boxes = median plus (red) range of dynamic force F_dyn_ (values as in Fig 5). ‘F_0_’ = static force in same position without vibration. ‘F_stat_>F_0_ %’ = Excess of F_stat_ above F_0_ as a percent of F_0_. ‘F_dyn_/F_stat_ %’ = dynamic force F_dyn_ as a percent of static force F_stat_. ‘Hz’ = platform frequency. ‘mm’ = platform stroke.

Fig 8 shows the preload F_stat_ in the knee/hip (green/blue columns) and the dynamic force F_dyn_ (yellow top boxes, values as in Fig 5) at different frequencies f (Hz) and strokes s (mm) on the Galileo and the Powerplate. The subjects always stood with 15° knee flexion. F_0_ are the static forces in the same position, but without vibrations. Added are the peak forces F_peak_ during walking (orange columns). For each f/s-condition, the excess of F_stat_ above F_0_ and the relation F_dyn_/F_stat_ are indicated as a percentage.

#### Static forces F_stat_

On the *Galileo*, F_stat_ with vibrations was higher than F_0_ without vibrations under three of four f/s-conditions (black percent numbers). These increases were +8% to +17% (knee) and +9% to +37% (hip). Under one condition each, different for the knee and hip, the static force was not influenced by the vibrations. F_stat_ was always smaller than 67%/52% (knee/hip) of the peak force F_peak_ during walking.

On the *Powerplate*, F_stat_ with vibrations mostly lay below F_0_ without vibrations (black percent numbers). It decreased by -7% to -16% (knee) and by -4% to -10% (hip). Under one condition, a small increase of +7% was observed in the hip. F_stat_ never exceeded 53%/40% (knee/hip) of F_peak_ during walking.

#### Relations between dynamic and static forces

On the *Galileo*, the dynamic force F_dyn_ never exceeded 22.6% of the static preload F_stat_ (green percentages). F_dyn_ was 9.8% to 22.6% (knee) and 10.3% to 16.4% (hip) of F_stat_. The force maximum F_stat_ + F_dyn_ with vibrations remained below 79%/56% (knee/hip) of F_peak_ during walking.

On the *Powerplate*, the vibration-induced *relative* force increases in the knee were similar to those produced on the Galileo, but in the hip these increases were much smaller. F_dyn_ values were 14.7% to 27.5% (knee) and 5.3% to 10.0% (hip) of F_stat_. The force maxima F_stat_ + F_dyn_ never exceeded 61%/44% (knee/hip) of F_peak_ during walking.

### Relation between increases in contact and muscle forces

The biomechanical knee model shows (Fig 3B and 3C) that the magnitude of the muscle force Q + A1 rises due to WBV by the same percentage as the contact force F if antagonistic muscle activities are lacking. Both increases are then proportional to the relative, vibration-induced increase in the ground reaction force W. If antagonistic activities exist in the assumed extent (Fig 3D and 3E), the muscle force Q + A1 increases only slightly more (+20%) than the contact forces F (+17%). The difference between both increases remains small if the lever arms of W, A1 and A2 are modified within realistic ranges, if the magnitudes of the antagonistic forces A1 and A2 are changed or if the ground reaction force W increases by more than 25%.

## Discussion

### Individual differences of dynamic force

As the large ranges in Figs 5 and 7 indicate, the dynamic force F_dyn_ differed substantially between the subjects. These large variations under the same conditions had the consequence that the changes in F_dyn_, caused by modifications of f, s, platform type or body position, were mostly non-significant. Therefore, the presented dynamic forces can only show trends. Different absolute values of F_dyn_ must be expected if other cohorts are investigated.

As the literature review (S1 File) revealed, very controversial results of WBV training were reported for all applications to the lower limbs. Positive, negative and absent influences on muscles, movements, EMG activities, bone remodelling, joint implants and osteoarthritis were published. The extreme dependency of the dynamic forces on the investigated subjects and vibration conditions, reported here, can probably explain many of these discrepancies.

### Other observations

F_dyn_ sometimes changed throughout the vibration time (example in Fig 4B). We speculate that these changes are caused by modifying the damping to reduce the discomfort of the exercise, especially in the knee joint. Unfortunately, we could not systematically investigate the underlying mechanical and physiological mechanisms because the elderly subjects were unwilling to extend the measurements further. Therefore, our data just describe the effects of WBW ‘as they are in practice’ and the underlying mechanisms still remain to be detected.

When standing on the forefeet on the GalileoS, the dynamic force F_dyn_ in the knee was 47% higher than the dynamic force F_foot_ at the foot. An explanation for this effect could not be found; possibly, resonance phenomena may explain this observation.

### Can contact forces in implants be transferred to natural joints?

The biomechanical conditions at the knee and hip are influenced, among other factors, by bone anatomy, muscle morphology and function. Depending on the surgical approach, certain muscles may become less functional, and others have to compensate for this loss of function. The anatomy may have been slightly changed, e.g., by implant neck length and anteversion.

A meta study [44] found the following main differences during walking between healthy subjects and subjects with hip replacement: reductions in walking velocity, stride length, range of motion and peak hip abductor moment. Obviously, the reductions of the last three parameters at least partially depend on the lowered walking speed. For a static position, as during WBV, the changes due to joint replacement will be of lower importance. We therefore assume that the contact forces observed in our cohorts are similar to those in healthy subjects.

### Fluctuations of the static force

Frequent fluctuations of the static force F_stat_ throughout the vibration time can have two causes: Either the subjects slightly changed their position or they applied additional, antagonistic muscle forces without position changes. Based on the average relation between knee contact forces and the knee flexion angle during squat [11], a rise of F_dyn_ by 60%BW, as in Fig 4C, would be associated with a flexion increase by 23°. This change is much larger than the supervising physiotherapist would have overlooked. Such strong fluctuations are therefore probably caused by changing antagonistic muscle activities, but weight shifts between both legs cannot be excluded.

### Do increased EMG signals indicate a proportional rise of muscle forces?

At a_foot_ = 50 m/s^2^, the EMG signals in the lower limbs were reported to increase by 100% [17]. Our data, however, showed (Fig 8) that the dynamic knee contact forces F_dyn_ at the same acceleration (25 Hz, 4 mm) increased much less, namely, by only 22.6% (Galileo) or 15.2% (Powerplate) of F_stat_. The biomechanical knee model (Fig 3) predicts that the muscle force rises by *nearly* the same percentage. These data indicate that the muscle forces rise much less in response to WBV than do the EMG signals.

Under *isometric* conditions, the relations between muscle forces and EMG signals are nearly linear, at least for the sum of normalized EMG signals from all active muscles that act across a joint [45]. However, the same publication also shows that the maximum possible force of a muscle *strongly* decreases if its shortening velocity is high. We assume that the observed discrepancy between the estimated dynamic muscle force increases and the reported rises of EMG signals may be at least partially due to this phenomenon. Muscle contractions during whole body vibration exercises are obviously not isometric, even if the body position is kept unchanged.

When using inverse dynamics and optimization methods to *calculate* muscle and contact forces during *walking* [46], the obtained *time courses* of muscle forces and additionally measured EMG signals agreed well, and the relation between *absolute* muscle and contact forces was nearly proportional. Similarly, as predicted by our biomechanical model for the *stance*, the contact forces were always higher than the muscle forces. However, no model for the relation between EMG and muscle forces was used in our study. The discrepancy between our estimated muscle forces and the EMG signals, measured by others [17], let us conclude that the models used for estimating muscle forces from EMG signals during walking [47, 48] cannot be applied in WBV studies.

### Can the dynamic forces be responsible for the positive effects of WBV?

The contact forces during WBV were only 5.3% to 27.5% higher than the forces measured in the same body positions without vibrations. The biomechanical model predicts that the muscle forces are increased by nearly the same amount. The sum of static plus dynamic forces during WBV was always below 79%/56% (knee/hip) of the peak forces during walking, an activity which is typical for frequent everyday activities [10, 11]. Both contact and muscle forces are therefore much smaller during WBV than during normal life. As compiled in S1 File, the outcome of WBV training is very controversial for all types of application to the lower limbs. The only small, vibration-induced force increases, reported here, indicate that the dynamic muscle forces *magnitudes* are probably not the cause of any positive muscle training effect of WBV.

*Other* effects may be decisive, for example simply the presence of vibrations independent of the dynamic force magnitudes, as shown in several studies: Bone reacted to *low-level* vibrations even in the absence of muscle activities [49, 50] or when the dynamic forces were much lower than the forces encountered during daily living [51, 52]. Identifying the complex reasons for positive, absent or negative influences of WBV on muscles and bone obviously requires innovative future hypotheses and investigations. Furthermore, the relation between the increases in muscle forces and EMG signals during WBV needs to be investigated in detail.

## Conclusions

- The influences of frequency, stroke, body position and platform type on the dynamic forces F_dyn_ in the knee and hip joints were often inconsistent. Increasing f or s, for example, sometimes nearly doubled F_dyn_ while leaving it unaffected in other cases. While increasing the knee flexion lowered F_dyn_ in knee and hip, the effect of standing in forefoot stance differed in knee and hip.
- The individual forces F_dyn_ differed appreciably under the same vibration conditions. Some subjects damped the vibration forces much better than others, perhaps because they were more motivated to do so or had different muscle tonus. On the Powerplate, F_dyn_ was sometimes even close to zero.
- Vibrations increased joint contact forces *and* muscle forces much less than the EMG signals would suggest. Our data indicate that predicting muscle forces from EMG signals during WBV is questionable.
- Damping of the vibration forces between the foot and knee was much less than that between the knee and hip. Determinations of F_dyn_ from platform accelerations are unreliable.
- Only a small and varying percentage of the *hypothetical* acceleration force of the platform (F’_foot_) really acted at the foot. This indicates the strong influence of damping in the body on the magnitude of F_dyn_.
- The Galileo, with alternating movements, generally produced larger dynamic forces than the Powerplate, with its parallel movements.

All these uncertainties together may be the reason that the effects of vibration training in different application fields (e.g., muscle training, bone remodelling or osteoarthritis) are described very controversially in the literature.

## Supporting information

S1 File Survey of literature on whole body vibration training. (DOCX)
