# Supplementary material for: Loading of the hip and knee joints during whole body vibration training
Source: PLoS One. 2018 Dec 12;13(12):e0207014. doi: 10.1371/journal.pone.0207014 (PMC6291191; doi:10.1371/journal.pone.0207014)
Supplement: S1 File — (DOCX) [file pone.0207014.s001.docx]

**Supplement 1**

**Survey of literature on whole body vibration training**

At least 560 scientific studies on whole body vibration (WBV) training have been published [1]. Only a small part of them could be checked and information on details, such as investigated frequencies, strokes and subjects, had to be omitted in the following survey, in which **Meta** **studies** (**MS**) are marked. The focus was set on WBV-induced changes in the *lower* extremities.

**Muscles and movements**

*Positive* *effects*: Improved knee extension, muscle strength, jump height and sit-to-stand performance were reported by MS [2-4]. Another study found similar positive influences [5]. In postmenopausal women, the muscle strength increased by 15% [6]. Training proved to be as effective as conventional training [7, 8], but possibly only at high accelerations [9]. In a bed rest study, WBV training proved to be positive for structure and function of the lower limb muscles [10].

*No or minor effects*: Low evidence for training effects was stated by four MS [11-14]. Other studies also observed no improvements [15-17]. In a bed rest study, the decrease in leg muscle volume was not prevented [18].

*Controversial effects:* Varying influences were reported in two MS [19, 20] and in other investigations [21, 22].

**EMG activities**

*Positive effects*: EMG activities were increased with WBV by factors of approximately 2.8 for the quadriceps femoris and between 2.8 and 27.6 for eight different muscles involved in pelvic stability [23]. However, much lower increases of only 8% were also published [24]. At the foot acceleration a_foot_ ≈ 50 m/s^2^, the average EMG signals from six muscles in the lower leg rose by 100% [25]. Another study reported less linear relations between a_foot_ and EMG signals, but only for a_foot_ < 0.74 g in one subject and a_foot_ < 2.88 g in another [26].

*No or minor effects*: Only small or no effects were found in [24, 27].

**Bone remodelling**

*Positive* *effects:* In patients with rheumatoid arthritis, bone mineral density improved [28]. Remodelling of new bone increased at higher bone strains, caused by (assumed) higher JCF, but that was only shown at much lower frequencies than those used during WBV training [29-32]. The rate of bone remodelling depended of the frequency of loads [33], and even small but fast vibrations (a_foot_ = 0.3 g, f = 30 Hz) increased the bone mineral density [34]. Comparable effects on the mineral density in the proximal femur were seen as those seen with resistance exercises [35]. In postmenopausal women, the bone mineral density increased by 0.95% when accelerations up to 5g were applied [6]. In young women the bone mass in the femur rose by about 2% when vibrations of very low magnitude were applied, i.e. when the acceleration forces were much lower than the forces during daily living [36]. Similar results were obtained by [37].

*No or minor effects:* In a MS, mineral density in osteoporotic femoral necks was not changed by WBV [4]. No changes of BMD in the femoral neck and greater trochanter were observed in [38]. Bone resorption and formation markers were not affected in a bed rest study [39]. Trabecular bone in proximal mice tibia stayed unaffected [40]. No influence on mineral density in the hip region and on bone remodelling biomarkers was reported [41]. Only slight benefits were observed for osteoporosis in the trochanter region (MS) [42]. Only small effects on bone architecture were found in older adults (MS) [12]. Mineral density in the hip area improved only slightly in postmenopausal women, children and adolescents, but not in young adults (MS) [43].

*Controversial, small or lacking effects:* Increases of cortical but not trabecular bone were observed, but only during the first nine weeks [44]. Controversial influences on trabecular and compact bone in healthy and osteotomized mouse femora were published, depending on the frequency; but even ‘disruption of fracture healing’ was expected [45]. Application of vibrations in mice with inhibited muscle activities didn’t prevent bone loss [46].

**Implants**

*Positive effects:* In patients with knee and hip implants, WBV and resistance training caused comparably strong improvements of muscle strength, muscle activation and mobility. Adverse side effects were not observed, but longer follow-up studies were suggested [47]. Positive effects were also seen in patients with knee arthroplasty [48]. Enhanced osteogenesis and osseointegration were described at non-cemented interfaces of titanium implants in rats, especially for low accelerations and high frequencies [49-54]. Osteogenesis was enhanced in vitro in rabbits [49].

*Negative effects*: In an analytical study, the number of bone cement cracks at the interface to bone increased with the number of loading cycles [55]. Another analytical investigation showed that even medium-high cyclic loads increased the number of cement cracks and the stresses at the interface to bone [56]. It must be kept in mind that daily WBV (5 minutes, 50 Hz) would load knee and hip implants 4.6 times more often than normal walking [57]. An overview of various studies reported that micro cracks in cement are initiated by voids, caused by the cement mixing technique, and also by roentgen opaque barium sulphate particles [58].

**Osteoarthritis**

*Positive effects:* Pain, stiffness and function (MS) improved in patients with knee osteoarthritis [59, 60]. Beneficial influences were also published by others [28, 61-63]. The performance in a step test improved [64].

*No effects:* Pain, functional performance and muscle strength did not improve [65, 66]. Pain and performance in walking tests remained unaffected [64]. Knee osteoarthritis did not improve in mice [40].

**Recommendations for subjects with implants or osteoarthritis**

Many public health advice sites warn against using WBV training in subjects with joint implants or osteoarthritis [67, 68]. The producers of the training devices state the same warning on their web sites and in their training manuals. Galileo mentions the insufficient knowledge about the long term effects of WBV on implant fixation and cartilage in osteoarthritic joints [69, 70].

**Summary of literature**

The reports on all possible applications of WBV training are controversial. This controversy may be due to the differences between investigated subjects and applied vibration parameters (frequency, stroke, platform type). It may also be that other parameters and mechanisms were not identified yet. In none of the studies were proportional increases of EMG signals and muscle forces or joint contact forces JCF explicitly stated.

**References for Supplement 1**

1. https://www.ncbi.nlm.nih.gov/pubmed. "Whole Body Vibration Training" Search in Titel and Abstract March 26, 2018].

2. Osawa Y, Oguma Y, Ishii N. The effects of whole-body vibration on muscle strength and power: a meta-analysis. Journal of musculoskeletal & neuronal interactions. 2013;13(3):380-90. Epub 2013/08/31. PubMed PMID: 23989260.

3. Lau RW, Liao LR, Yu F, Teo T, Chung RC, Pang MY. The effects of whole body vibration therapy on bone mineral density and leg muscle strength in older adults: a systematic review and meta-analysis. Clinical rehabilitation. 2011;25(11):975-88.
Epub 2011/08/19. doi: 10.1177/0269215511405078. PubMed PMID: 21849376.

4. Ma C, Liu A, Sun M, Zhu H, Wu H. Effect of whole-body vibration on reduction of bone loss and fall prevention in postmenopausal women: a meta-analysis and systematic review. Journal of orthopaedic surgery and research. 2016;11:24.
 Epub 2016/02/19. doi: 10.1186/s13018-016-0357-2.
PubMed PMID: 26888467; PubMed Central PMCID: PMC4758089.

5. Osawa Y, Oguma Y. Effects of resistance training with whole-body vibration on muscle fitness in untrained adults. Scandinavian journal of medicine & science in sports. 2013;23(1):84-95.
Epub 2011/08/05. doi: 10.1111/j.1600-0838.2011.01352.x. PubMed PMID: 21812821.

6. Verschueren SM, Roelants M, Delecluse C, Swinnen S, Vanderschueren D, Boonen S. Effect of 6-month whole body vibration training on hip density, muscle strength, and postural control in postmenopausal women: a randomized controlled pilot study. Journal of bone and mineral research : the official journal of the American Society for Bone and Mineral Research. 2004;19(3):352-9.
Epub 2004/03/26. doi: 10.1359/JBMR.0301245. PubMed PMID: 15040822.

7. Delecluse C, Roelants M, Verschueren S. Strength increase after whole-body vibration compared with resistance training. Medicine and science in sports and exercise. 2003;35(6):1033-41. Epub 2003/06/05. doi: 10.1249/01.MSS.0000069752.96438.B0. PubMed PMID: 12783053.

8. Roelants M, Delecluse C, Verschueren SM. Whole-body-vibration training increases knee-extension strength and speed of movement in older women. Journal of the American Geriatrics Society. 2004;52(6):901-8.
Epub 2004/05/27. doi: 10.1111/j.1532-5415.2004.52256.x. PubMed PMID: 15161453.

9. Petit PD, Pensini M, Tessaro J, Desnuelle C, Legros P, Colson SS. Optimal whole-body vibration settings for muscle strength and power enhancement in human knee extensors. Journal of electromyography and kinesiology : official journal of the International Society of Electrophysiological Kinesiology. 2010;20(6):1186-95.
doi: 10.1016/j.jelekin.2010.08.002. PubMed PMID: 20801671.

10. Blottner D, Salanova M, Puttmann B, Schiffl G, Felsenberg D, Buehring B, et al. Human skeletal muscle structure and function preserved by vibration muscle exercise following 55 days of bed rest. European journal of applied physiology. 2006;97(3):261-71.
Epub 2006/03/29. doi: 10.1007/s00421-006-0160-6. PubMed PMID: 16568340.

11. Lindberg J, Carlsson J. The effects of whole-body vibration training on gait and walking ability - a systematic review comparing two quality indexes. Physiotherapy theory and practice. 2012;28(7):485-98.
Epub 2012/01/05. doi: 10.3109/09593985.2011.641670. PubMed PMID: 22214345.

12. Mikhael M, Orr R, Fiatarone Singh MA. The effect of whole body vibration exposure on muscle or bone morphology and function in older adults: a systematic review of the literature. Maturitas. 2010;66(2):150-7.
Epub 2010/02/23. doi: 10.1016/j.maturitas.2010.01.013. PubMed PMID: 20171817.

13. Yang J, Seo D. The effects of whole body vibration on static balance, spinal curvature, pain, and disability of patients with low back pain. Journal of physical therapy science. 2015;27(3):805-8. Epub 2015/05/02. doi: 10.1589/jpts.27.805.
PubMed PMID: 25931735; PubMed Central PMCID: PMC4395719.

14. Lu J, Xu G, Wang Y. Effects of whole body vibration training on people with chronic stroke: a systematic review and meta-analysis. Topics in stroke rehabilitation. 2015;22(3):161-8.
Epub 2015/06/19. doi: 10.1179/1074935714Z.0000000005. PubMed PMID: 26084320.

15. de Ruiter CJ, Van Raak SM, Schilperoort JV, Hollander AP, de Haan A. The effects of 11 weeks whole body vibration training on jump height, contractile properties and activation of human knee extensors. European journal of applied physiology. 2003;90(5-6):595-600.
Epub 2003/08/19. doi: 10.1007/s00421-003-0931-2. PubMed PMID: 12923646.

16. Osawa Y, Oguma Y, Onishi S. Effects of whole-body vibration training on bone-free lean body mass and muscle strength in young adults. Journal of sports science & medicine. 2011;10(1):97-104. Epub 2011/01/01.
PubMed PMID: 24149301; PubMed Central PMCID: PMC3737898.

17. Hortobagyi T, Lesinski M, Fernandez-Del-Olmo M, Granacher U. Small and inconsistent effects of whole body vibration on athletic performance: a systematic review and meta-analysis. European journal of applied physiology. 2015;115(8):1605-25.
Epub 2015/06/04. doi: 10.1007/s00421-015-3194-9.
PubMed PMID: 26037127; PubMed Central PMCID: PMC4503864.

18. Zange J, Mester J, Heer M, Kluge G, Liphardt AM. 20-Hz whole body vibration training fails to counteract the decrease in leg muscle volume caused by 14 days of 6 degrees head down tilt bed rest. European journal of applied physiology. 2009;105(2):271-7. Epub 2008/10/31. doi: 10.1007/s00421-008-0899-z. PubMed PMID: 18972127.

19. Lam FM, Lau RW, Chung RC, Pang MY. The effect of whole body vibration on balance, mobility and falls in older adults: a systematic review and meta-analysis. Maturitas. 2012;72(3):206-13.
Epub 2012/05/23. doi: 10.1016/j.maturitas.2012.04.009. PubMed PMID: 22609157.

20. Orr R. The effect of whole body vibration exposure on balance and functional mobility in older adults: a systematic review and meta-analysis. Maturitas. 2015;80(4):342-58.
Epub 2015/01/30. doi: 10.1016/j.maturitas.2014.12.020. PubMed PMID: 25631348.

21. Cloak R, Lane A, Wyon M. Professional Soccer Player Neuromuscular Responses and Perceptions to Acute Whole Body Vibration Differ from Amateur Counterparts. Journal of sports science & medicine. 2016;15(1):57-64. Epub 2016/03/10.
PubMed PMID: 26957927; PubMed Central PMCID: PMC4763847.

22. Bush JA, Blog GL, Kang J, Faigenbaum AD, Ratamess NA. Effects of quadriceps strength after static and dynamic whole-body vibration exercise. Journal of strength and conditioning research / National Strength & Conditioning Association. 2015;29(5):1367-77. Epub 2014/10/01. doi: 10.1519/JSC.0000000000000709. PubMed PMID: 25268289.

23. Kim JH, Seo HJ. Influence of pelvic position and vibration frequency on muscle activation during whole body vibration in quiet standing. Journal of physical therapy science. 2015;27(4):1055-8. Epub 2015/05/23. doi: 10.1589/jpts.27.1055.
PubMed PMID: 25995555; PubMed Central PMCID: PMC4433976.

24. Lienhard K, Vienneau J, Friesenbichler B, Nigg S, Meste O, Nigg BM, et al. The Effect of Whole-body Vibration on Muscle Activity in Active and Inactive Subjects. International journal of sports medicine. 2015;36(7):585-91.
Epub 2015/03/12. doi: 10.1055/s-0034-1398650. PubMed PMID: 25760148.

25. Lienhard K, Vienneau J, Nigg S, Friesenbichler B, Nigg BM. Older adults show higher increases in lower-limb muscle activity during whole-body vibration exercise. Journal of biomechanics. 2017;52:55-60.
doi: 10.1016/j.jbiomech.2016.12.009. PubMed PMID: WOS:000395217300009.

26. Di Giminiani R, Masedu F, Padulo J, Tihanyi J, Valenti M. The EMG activity-acceleration relationship to quantify the optimal vibration load when applying synchronous whole-body vibration. Journal of electromyography and kinesiology : official journal of the International Society of Electrophysiological Kinesiology. 2015;25(6):853-9.
Epub 2015/10/08. doi: 10.1016/j.jelekin.2015.09.004. PubMed PMID: 26443890.

27. Avelar NC, Ribeiro VG, Mezencio B, Fonseca SF, Tossige-Gomes R, da Costa SJ, et al. Influence of the knee flexion on muscle activation and transmissibility during whole body vibration. Journal of electromyography and kinesiology : official journal of the International Society of Electrophysiological Kinesiology. 2013;23(4):844-50.
Epub 2013/05/07. doi: 10.1016/j.jelekin.2013.03.014. PubMed PMID: 23643467.

28. Prioreschi A, Makda MA, Tikly M, McVeigh JA. In Patients with Established RA, Positive Effects of a Randomised Three Month WBV Therapy Intervention on Functional Ability, Bone Mineral Density and Fatigue Are Sustained for up to Six Months. PloS one. 2016;11(4):e0153470. Epub 2016/04/14. doi: 10.1371/journal.pone.0153470.
PubMed PMID: 27073832; PubMed Central PMCID: PMC4830593.

29. Carter DR, Van Der Meulen MC, Beaupre GS. Mechanical factors in bone growth and development. Bone. 1996;18(1 Suppl):5S-10S. Epub 1996/01/01. PubMed PMID: 8717541.

30. Robling AG, Castillo AB, Turner CH. Biomechanical and molecular regulation of bone remodeling. Annual review of biomedical engineering. 2006;8:455-98. Epub 2006/07/13. doi: 10.1146/annurev.bioeng.8.061505.095721. PubMed PMID: 16834564.

31. Robling AG, Duijvelaar KM, Geevers JV, Ohashi N, Turner CH. Modulation of appositional and longitudinal bone growth in the rat ulna by applied static and dynamic force. Bone. 2001;29(2):105-13. Epub 2001/08/15. PubMed PMID: 11502470.

32. Turner CH. Three rules for bone adaptation to mechanical stimuli. Bone. 1998;23(5):399-407. Epub 1998/11/21. PubMed PMID: 9823445.

33. Turner CH, Owan I, Takano Y. Mechanotransduction in bone: role of strain rate. The American journal of physiology. 1995;269(3 Pt 1):E438-42.
Epub 1995/09/01. PubMed PMID: 7573420.

34. Rubin C, Turner AS, Mallinckrodt C, Jerome C, McLeod K, Bain S. Mechanical strain, induced noninvasively in the high-frequency domain, is anabolic to cancellous bone, but not cortical bone. Bone. 2002;30(3):445-52.
Epub 2002/03/08. PubMed PMID: 11882457.

35. Zaki ME. Effects of whole body vibration and resistance training on bone mineral density and anthropometry in obese postmenopausal women. Journal of osteoporosis. 2014;2014:702589. Epub 2014/08/20. doi: 10.1155/2014/702589.
PubMed PMID: 25136473; PubMed Central PMCID: PMC4086652.

36. Gilsanz V, Wren TA, Sanchez M, Dorey F, Judex S, Rubin C. Low‐level, high‐frequency mechanical signals enhance musculoskeletal development of young women with low BMD. Journal of Bone and Mineral Research. 2006;21(9):1464-74.

37. Rubin C, Recker R, Cullen D, Ryaby J, McCabe J, McLeod K. Prevention of postmenopausal bone loss by a low‐magnitude, high‐frequency mechanical stimuli: a clinical trial assessing compliance, efficacy, and safety. Journal of Bone and Mineral Research. 2004;19(3):343-51.

38. Cidem M, Karacan I, Diracoglu D, Yildiz A, Kucuk SH, Uludag M, et al. A Randomized Trial on the Effect of Bone Tissue on Vibration-induced Muscle Strength Gain and Vibration-induced Reflex Muscle Activity. Balkan medical journal. 2014;31(1):11-22. Epub 2014/09/11. doi: 10.5152/balkanmedj.2013.9482.
PubMed PMID: 25207162; PubMed Central PMCID: PMC4115987.

39. Baecker N, Frings-Meuthen P, Heer M, Mester J, Liphardt AM. Effects of vibration training on bone metabolism: results from a short-term bed rest study. European journal of applied physiology. 2012;112(5):1741-50.
Epub 2011/09/07. doi: 10.1007/s00421-011-2137-3. PubMed PMID: 21894450.

40. McCann MR, Patel P, Pest MA, Ratneswaran A, Lalli G, Beaucage KL, et al. Repeated exposure to high-frequency low-amplitude vibration induces degeneration of murine intervertebral discs and knee joints. Arthritis Rheumatol. 2015;67(8):2164-75.
Epub 2015/04/22. doi: 10.1002/art.39154. PubMed PMID: 25891852.

41. Kiel DP, Hannan MT, Barton BA, Bouxsein ML, Sisson E, Lang T, et al. Low-Magnitude Mechanical Stimulation to Improve Bone Density in Persons of Advanced Age: A Randomized, Placebo-Controlled Trial. Journal of bone and mineral research : the official journal of the American Society for Bone and Mineral Research. 2015;30(7):1319-28. Epub 2015/01/13. doi: 10.1002/jbmr.2448.
PubMed PMID: 25581217; PubMed Central PMCID: PMC4834704.

42. Oliveira LC, Oliveira RG, Pires-Oliveira DA. Effects of whole body vibration on bone mineral density in postmenopausal women: a systematic review and meta-analysis. Osteoporosis international : a journal established as result of cooperation between the European Foundation for Osteoporosis and the National Osteoporosis Foundation of the USA. 2016.
Epub 2016/05/06. doi: 10.1007/s00198-016-3618-3. PubMed PMID: 27145947.

43. Slatkovska L, Alibhai SM, Beyene J, Cheung AM. Effect of whole-body vibration on BMD: a systematic review and meta-analysis. Osteoporosis international : a journal established as result of cooperation between the European Foundation for Osteoporosis and the National Osteoporosis Foundation of the USA. 2010;21(12):1969-80.
Epub 2010/04/22. doi: 10.1007/s00198-010-1228-z. PubMed PMID: 20407890.

44. Gnyubkin V, Guignandon A, Laroche N, Vanden-Bossche A, Malaval L, Vico L. High-acceleration whole body vibration stimulates cortical bone accrual and increases bone mineral content in growing mice. Journal of biomechanics. 2016.
Epub 2016/05/15. doi: 10.1016/j.jbiomech.2016.04.031. PubMed PMID: 27178020.

45. Wehrle E, Wehner T, Heilmann A, Bindl R, Claes L, Jakob F, et al. Distinct frequency dependent effects of whole-body vibration on non-fractured bone and fracture healing in mice. J Orthop Res. 2014;32(8):1006-13.
Epub 2014/04/15. doi: 10.1002/jor.22629. PubMed PMID: 24729351.

46. Manske SL, Good CA, Zernicke RF, Boyd SK. High-frequency, low-magnitude vibration does not prevent bone loss resulting from muscle disuse in mice following botulinum toxin injection. PloS one. 2012;7(5):e36486.

47. Johnson AW, Myrer JW, Hunter I, Feland JB, Hopkins JT, Draper DO, et al. Whole-body vibration strengthening compared to traditional strengthening during physical therapy in individuals with total knee arthroplasty. Physiotherapy theory and practice. 2010;26(4):215-25.
Epub 2010/04/20. doi: 10.3109/09593980902967196. PubMed PMID: 20397856.

48. Bily W, Franz C, Trimmel L, Loefler S, Cvecka J, Zampieri S, et al. Effects of Leg-Press Training With Moderate Vibration on Muscle Strength, Pain, and Function After Total Knee Arthroplasty: A Randomized Controlled Trial. Archives of physical medicine and rehabilitation. 2016.
Epub 2016/01/15. doi: 10.1016/j.apmr.2015.12.015. PubMed PMID: 26763947.

49. Jing D, Tong S, Zhai M, Li X, Cai J, Wu Y, et al. Effect of low-level mechanical vibration on osteogenesis and osseointegration of porous titanium implants in the repair of long bone defects. Scientific reports. 2015;5:17134.
Epub 2015/11/26. doi: 10.1038/srep17134.
PubMed PMID: 26601709; PubMed Central PMCID: PMC4658533.

50. Liang YQ, Qi MC, Xu J, Liu HW, Dong W, Li JY, et al. Low-magnitude high-frequency loading, by whole-body vibration, accelerates early implant osseointegration in ovariectomized rats. Molecular medicine reports. 2014;10(6):2835-42.
Epub 2014/10/02. doi: 10.3892/mmr.2014.2597.
PubMed PMID: 25270245; PubMed Central PMCID: PMC4227418.

51. Ogawa T, Vandamme K, Zhang XL, Naert I, Possemiers T, Chaudhari A, et al. Stimulation of Titanium Implant Osseointegration Through High-Frequency Vibration Loading is Enhanced when Applied at High Acceleration. Calcified tissue international. 2014;95(5):467-75. doi: 10.1007/s00223-014-9896-x.
PubMed PMID: ISI:000343088500010.

52. Zhou Y, Guan X, Liu T, Wang X, Yu M, Yang G, et al. Whole body vibration improves osseointegration by up-regulating osteoblastic activity but down-regulating osteoblast-mediated osteoclastogenesis via ERK1/2 pathway. Bone. 2015;71:17-24.
Epub 2014/10/12. doi: 10.1016/j.bone.2014.09.026. PubMed PMID: 25304090.

53. Chatterjee M, Hatori K, Duyck J, Sasaki K, Naert I, Vandamme K. High-frequency loading positively impacts titanium implant osseointegration in impaired bone. Osteoporosis Int. 2015;26(1):281-90.
doi: 10.1007/s00198-014-2824-0. PubMed PMID: ISI:000347689400030.

54. Chen BL, Li YQ, Xie DH, Yang XX. Low-magnitude high-frequency loading via whole body vibration enhances bone-implant osseointegration in ovariectomized rats. J Orthop Res. 2012;30(5):733-9. doi: 10.1002/jor.22004. PubMed PMID: ISI:000301232700010.

55. Waanders D, Janssen D, Mann KA, Verdonschot N. The behavior of the micro-mechanical cement-bone interface affects the cement failure in total hip replacement. Journal of biomechanics. 2011;44(2):228-34.
Epub 2010/11/03. doi: 10.1016/j.jbiomech.2010.10.020.
PubMed PMID: 21036358; PubMed Central PMCID: PMC3019267.

56. Waanders D, Janssen D, Mann KA, Verdonschot N. The effect of cement creep and cement fatigue damage on the micromechanics of the cement-bone interface. Journal of biomechanics. 2010;43(15):3028-34.
Epub 2010/08/10. doi: 10.1016/j.jbiomech.2010.06.031.
PubMed PMID: 20692663; PubMed Central PMCID: PMC2975749.

57. Morlock M, Schneider E, Bluhm A, Vollmer M, Bergmann G, Muller V, et al. Duration and frequency of every day activities in total hip patients. Journal of biomechanics. 2001;34(7):873-81. Epub 2001/06/19. PubMed PMID: 11410171.

58. Shearwood-Porter N, Browne M, Sinclair I. Micromechanical characterisation of failure in acrylic bone cement: the effect of barium sulphate agglomerates. Journal of the mechanical behavior of biomedical materials. 2012;13:85-92.
Epub 2012/07/31. doi: 10.1016/j.jmbbm.2012.04.012. PubMed PMID: 22842279.

59. Wang P, Yang X, Yang Y, Yang L, Zhou Y, Liu C, et al. Effects of whole body vibration on pain, stiffness and physical functions in patients with knee osteoarthritis: a systematic review and meta-analysis. Clinical rehabilitation. 2015;29(10):939-51.
Epub 2014/12/20. doi: 10.1177/0269215514564895. PubMed PMID: 25525066.

60. Zafar H, Alghadir A, Anwer S, Al-Eisa E. Therapeutic effects of whole-body vibration training in knee osteoarthritis: a systematic review and meta-analysis. Archives of physical medicine and rehabilitation. 2015;96(8):1525-32.
Epub 2015/04/02. doi: 10.1016/j.apmr.2015.03.010. PubMed PMID: 25827655.

61. Tossige-Gomes R, Avelar NC, Simao AP, Neves CD, Brito-Melo GE, Coimbra CC, et al. Whole-body vibration decreases the proliferativeb response of TCD4(+) cells in elderly individuals with knee osteoarthritis. Brazilian journal of medical and biological research = Revista brasileira de pesquisas medicas e biologicas / Sociedade Brasileira de Biofisica [et al]. 2012;45(12):1262-8. Epub 2012/09/06.
PubMed PMID: 22948377; PubMed Central PMCID: PMC3854226.

62. Segal NA, Glass NA, Shakoor N, Wallace R. Vibration Platform Training in Women at Risk for Symptomatic Knee Osteoarthritis. Pm&R. 2013;5(3):201-9.
doi: 10.1016/j.pmrj.2012.07.011. PubMed PMID: ISI:000316433300007.

63. Tsuji T, Yoon J, Aiba T, Kanamori A, Okura T, Tanaka K. Effects of whole-body vibration exercise on muscular strength and power, functional mobility and self-reported knee function in middle-aged and older Japanese women with knee pain. The Knee. 2014;21(6):1088-95.
Epub 2014/08/26. doi: 10.1016/j.knee.2014.07.015. PubMed PMID: 25153612.

64. Salmon JR, Roper JA, Tillman MD. Does Acute Whole-Body Vibration Training Improve the Physical Performance of People with Knee Osteoarthritis? Journal of Strength and Conditioning Research. 2012;26(11):2983-9.
doi: 10.1519/JSC.0b013e318242a4be. PubMed PMID: ISI:000310569900010.

65. Li X, Wang XQ, Chen BL, Huang LY, Liu Y. Erratum to "Whole-Body Vibration Exercise for Knee Osteoarthritis: A Systematic Review and Meta-Analysis". Evidence-based complementary and alternative medicine : eCAM. 2015;2015:636435.
Epub 2015/12/08. doi: 10.1155/2015/636435.
PubMed PMID: 26640500; PubMed Central PMCID: PMC4660018.

66. Li X, Wang XQ, Chen BL, Huang LY, Liu Y. Whole-Body Vibration Exercise for Knee Osteoarthritis: A Systematic Review and Meta-Analysis. Evidence-based complementary and alternative medicine : eCAM. 2015;2015:758147.
Epub 2015/09/09. doi: 10.1155/2015/758147.
PubMed PMID: 26347287; PubMed Central PMCID: PMC4540999.

67. http://westhartfordchiropractic.com. Whole Body Vibration FAQ's2016 May 22, 2016. Available from: http://westhartfordchiropractic.com/wave-whole-body-vibration/whole-body-vibration-faq-s.html.

68. http://www.bcvibranthealth.com. Whole Body Vibration—Contraindications2016 May 22, 2016. Available from: http://www.bcvibranthealth.com/whole-body-vibration/contraindications.php.

69. https://www.galileo-training.com. Galileo training: contraindications2016. Available from: https://www.galileo-training.com/de-english/products/galileo-therapy-systems/background/contraindications.html.

70. https://www.hypervibe.com. Is Whole Body Vibration Safe?2016 May 22, 2016. Available from: https://www.hypervibe.com/us/vibration_machine_contraindications.php.
